# Partial Replacement of Ground Corn with Glycerol in Beef Cattle Diets: Intake, Digestibility, Performance, and Carcass Characteristics

**DOI:** 10.1371/journal.pone.0148224

**Published:** 2016-01-28

**Authors:** Pedro Del Bianco Benedeti, Pedro Veiga Rodrigues Paulino, Marcos Inácio Marcondes, Ivan França Smith Maciel, Matheus Custódio da Silva, Antonio Pinheiro Faciola

**Affiliations:** 1 Department of Animal Sciences, Federal University of Viçosa, Viçosa, Minas Gerais, Brazil; 2 Cargill Animal Nutrition / Nutron, Campinas, São Paulo, Brazil; 3 Department of Agriculture, Nutrition, and Veterinary Science, University of Nevada, Reno, Nevada, United States of America; Wageningen UR Livestock Research, NETHERLANDS

## Abstract

The objective of this study was to evaluate the effects of replacing dry ground corn with crude glycerol on intake, apparent digestibility, performance, and carcass characteristics of finishing beef bulls. A completely randomized block design experiment with 25 d for adaptation and 100 d for data collection was conducted, in which 3,640 Nellore bulls (367 ± 36.8 kg; 18 ± 3 mo) were blocked by body weight and assigned to 20 pens. Bulls were randomly assigned to one of four treatments: 0, 5, 10, and 15% (dry matter basis) of crude glycerol in the diet. Initially, 20 bulls were slaughtered to serve as a reference to estimate initial empty body weight, which allowed for carcass gain calculation. Bulls were weighed at the beginning, at two-thirds, and at the end of the experiment for performance calculations. Carcass measurements were obtained by ultrasound. Fecal output was estimated using indigestible neutral detergent fiber as an internal marker. Data were analyzed using the mixed procedures in SAS 9.2 (SAS Institute Inc., Cary, NC). Intake of dry matter, organic matter, and neutral detergent fiber decreased linearly (*P* < 0.05) with crude glycerol inclusion. However, crude glycerol levels did not affect (*P* > 0.05) intakes of crude protein, non-fiber carbohydrates, and total digestible nutrients. Digestibility of dry matter, organic matter, neutral detergent fiber, and total digestible nutrients increased quadratically (*P* < 0.05) with the inclusion of crude glycerol in the diet. Crude glycerol inclusion did not change the intake of digestible dry matter, average daily gain, final body weight, carcass gain, carcass dressing, gain-to-feed ratio, *Longissimus thoracis* muscle area, and back and rump fat thicknesses (*P* > 0.05). These results suggest that crude glycerol may be included in finishing beef diets at levels up to 15% without impairing performance and carcass characteristics.

## Introduction

Corn is typically the main feed ingredient used for finishing cattle in feedlots [1]. However, due to its high cost, alternative energy sources may have the potential to improve livestock profitability. The growth of the biodiesel industry worldwide has increased the availability of low cost crude glycerol (**CG**), in Brazil alone, it has been estimated that in 2014 the country produced 3.42 billion liters of biodiesel, yielding 341 million liters of CG [2]. This may position CG as a viable alternative feed source for finishing cattle. The CG primary component is glycerol, which has an estimated metabolic energy of 4.03 Mcal/kg [3], a higher value than corn starch [4]. In the rumen, glycerol is fermented to propionate [5,6], main gluconeogenic precursor for ruminant animals [7]. Furthermore, the glycerol that escapes rumen fermentation may be converted to glucose in the liver [7]. Therefore, from both economic and energy perspectives, CG has the potential to partially replace corn as an alternative energy source for beef cattle finishing diets.

Some research has been done on the use of CG by ruminants [8,9]; however, the effects of CG have been conflicting and the maximum levels of CG in the diet of finishing cattle have not been established. Discrepancies across experiments may be due to the limited number of experimental units used among other aspects; therefore, it is relevant to evaluate the effects of CG using a large number of animals.

The objective of this study was to evaluate the effects of replacing corn with CG in the diets of 3,640 Nellore bulls finished in feedlot. We hypothesized that CG may partially replace dry ground corn (**DGC**) as dietary energy source in the diet of finishing cattle diets and may be included at concentrations up to 15% [dry matter (**DM**) basis] without compromising intake, apparent digestibility, performance, and carcass characteristics.

## Materials and Methods

### Ethics Statement

Care and handling of all experimental animals were conducted under protocols approved by the Institutional Animal Care and Use Committee of the Animal Science Department of the Federal University of Viçosa, protocol number 84/2013.

### Animals, Experimental Design, and Diet Composition

A total of 3,640 Nellore bulls averaging [body weight (**BW**) = 367.0 ± 36.8 kg and 18 ± 3 mo] were allocated to 20 pens (182 animals/pen; 16.3 m^2^/animal) in a commercial feedlot located at Ribas do Rio Pardo, MS, Brazil (21° 9'16.15"S, 53°16'46.85"W, elev. 348 m); all pens were equipped with water and feed troughs. Before the beginning of the experiment, all bulls were weighed, vaccinated, dewormed, and received individual numbered tags. Cattle were adapted to the diets, facilities, and management for 25 d. Then, 20 bulls (one per pen) were randomly selected and slaughtered to serve as a reference for initial empty BW and initial carcass dressing. After the adaptation period the animals remained for an additional 100 d in the trial.

Bulls were blocked by BW into four blocks of 905 animals with similar BW and then within each block, bulls were randomly assigned to one of four experimental treatments in a completely randomized block design resulting in 905 animals per treatment. Animals were allocated to 20 pens (181 animals per pen and five pens per treatment).

Experimental treatments consisted of four dietary levels of CG; 0, 5, 10, and 15% (DM basis) as a substitute of DGC. Experimental diets were composed of 15.3% of corn silage and 84.6% of concentrate (DM basis) and were formulated to meet nutritional requirements of beef cattle [10]. Ingredient and chemical composition of the experimental diets are presented in Table 1. The CG chemical analyses were conducted using the esterification process of vegetable oils with subsequent purification according to the standards established by the Ministry of Agriculture, Livestock and Food Supply [11], CG was composed of 82.8% glycerol, 8.4% water, 6.3% ash, 1.4% fatty acids, 1.1% crude protein (**CP**), and 0.01% methanol.

**Table 1:** Ingredient and chemical composition of experimental diets.

Item	Crude glycerol, %			
	0	5	10	15
Ingredient, % DM^1^				
Corn silage	15.35	15.35	15.35	15.35
Dry ground corn	38.89	33.72	28.56	23.40
Crude glycerol^2^	0.00	5.00	10.00	15.00
Citrus pulp	25.00	25.00	25.00	25.00
Cottonseed cake	16.58	16.58	16.58	16.58
Urea	0.80	0.97	1.13	1.29
Slow release urea^3^	0.45	0.45	0.45	0.45
Vitamin-mineral premix^4^	2.93	2.93	2.93	2.93
Composition, % DM				
Dry matter, %	80.2	80.3	80.3	80.3
Non-fiber carbohydrates^5^	48.0	48.8	49.6	50.4
Neutral detergent fiber	28.4	27.7	27.0	26.2
Crude protein	14.7	14.8	14.8	14.9
Ash	6.8	7.0	7.3	7.6
Ether extract	4.4	4.2	4.1	3.9

^1^DM = dry matter.

^2^The crude glycerol used in this study was analyzed and contained 82.8% glycerol, 8.4% water, 6.3% ash, 1.4% fatty acids, 1.1% crude protein, and 0.01% methanol. It was obtained from an esterification process of vegetable oils with subsequent purification. It was provided by Granol Indústria Comércio e Exportação S.A (Cachoeira do Sul, RS, Brazil) and met the standards established by the Ministry of Agriculture, Livestock and Supply [11].

^3^Optigen 1200 controlled-release nitrogen, Alltech, Araucária, PR, Brazil.

^4^Provided (per kg of DM): 195 g of Ca, 50 g of Na, 26.7 g of S, 20 g of P, 17 g of Mg, 2,000 mg of Zn, 1,000 mg of monensin, 840 mg of Mn, 490 mg of Fe, 420 mg of Cu, 25 mg of Co, 25 mg of I, 7 mg of Se, *Saccharomyces cerevisiae* 100 x 10^9^ CFU, 100,000 IU of vitamin A, 10,400 IU of vitamin D3, 242 IU of vitamin E.

^5^Non-fiber carbohydrates = 100 − [(crude protein–crude protein from urea + urea) + neutral detergent fiber + ether extract + Ash] [12].

### Experimental Procedures and Sample Collections

Bulls were fed four times daily at 0700, 1000, 1300, and 1600 h. Diets were mixed in two mixer feeder wagons (3142 Reel Auggie, Kuhn, Passo Fundo, RS, Brazil). The wagons were checked for residual feed between each dietary mix to avoid cross-contamination. Feed bunkers were evaluated at 0530h each day to quantify orts and to adjust daily feed allowance to a maximum of 5% orts. Samples of feed and orts were collected daily from each pen and then composited every 14 d. The samples were frozen at −18°C until further laboratory analysis.

Bulls were observed at least once daily during the experimental period to record the presence of anything abnormal (loss of tags, bloat, or injury) that may compromise the study and bulls that presented these conditions (n = 61) were removed from the experiment. Individual DM intake was calculated by the ratio between the amount of diet offered minus the orts per pen and the number of bulls per pen. Bulls were individually weighed after a 16-h solid-feed fasting at the beginning, at two-thirds, and at the end of the experiment. The average daily gain (**ADG**) was determined as the slope of the regression of BW. On the same day, measurements of the *Longissimus thoracis* muscle area (**LMA**), back fat thickness (**BFT**), and rump fat thickness (**RFT**) were obtained by ultrasound (Aloka Echo Camera Model SSD-500, Campinas, SP, Brazil). The LMA was measured on a transversal section in the 12^th^ rib, BFT was measured on a longitudinal section in the 12^th^ rib, and RFT was measured on a longitudinal section on the rump [13]. Ultrasound images were collected and analyzed by a certified technician (Ultrasound Guidelines Council) from the Aval Serviços Tecnológicos S/S, Goiania, GO, Brazil.

Apparent digestibility of nutrients was determined in two periods of three consecutive day, during the sampling collections (d 43–45 and d 87–89). Because collecting fecal samples from all 3,620 bulls would be unfeasible, on each sampling day, fecal samples from 50 animals per pen were taken, from the pen floor immediately after defecation and any dirt was carefully removed to avoid contamination. To ensure sample representation and homogeneity, all 50 samples were collected in the morning of the first day of collection, in the afternoon of the second day, and at night of the third day. This process was performed twice (total of 6 d); therefore, 300 fecal samples were collected per pen corresponding to 6,000 samples. Samples were composited by pen, homogenized, and 300 g (wet basis) were weighed and stored in plastic bags and frozen at −18°C. Fecal excretion was estimated using indigestible neutral detergent fiber (**NDF**) as an internal marker, which was obtained after a 12-d in situ incubation according to Huhtanen *et al*. [14]. The digestible DM intake was calculated as follow: [DM intake (kg) x DM digestibility (%)] / 100.

After the experimental period, animals were transported to a commercial abattoir (JBS–Friboi, Campo Grande, MS, Brazil) for slaughter. Pre-harvest handling was conducted in accordance with good animal welfare practices, and slaughtering procedures followed strict guidelines stablished and regulated by the Sanitary and Industrial Inspection Regulation for Animal Origin Products [15]. At the abattoir, hot carcasses were weighed individually and received scores by a certified technician for fat deposition according to the Brazilian system for classification of cattle carcasses [16], briefly, the carcass fatness was classified into five fat categories: thin (< 1 mm), scarce (1–3 mm), medium (3–6 mm), uniform (6–10 mm), and excessive (> 10 mm). This data was compared with ultrasonography evaluation to determine the accuracy between the fatness classification (from abattoir technician) and the ultrasound measurements. Carcass dressing was calculated based on the final carcass weight and BW ratio after fasting. Initial carcass weight was calculated using the value of 54.3% of live BW, which was obtained in the reference slaughter at beginning of the trial. The carcass ADG was determined considering the difference between final and initial carcass weights.

### Chemical Analysis

Feed, orts, and fecal samples were thawed, oven dried at 55°C for 48 h, and then ground through a 1-mm screen in a Wiley mill (Arthur H. Thomas, Philadelphia, PA). After that, samples were analyzed for DM [17], ash (method 942.05; AOAC, 2005), CP (method 984.13; AOAC, 2005), and ether extract (**EE**; method 920.39; AOAC, 2005). The organic matter (**OM**) was calculated as the difference between DM and ash contents. For NDF analysis, samples were treated with alpha thermo-stable amylase without sodium sulfite according to Van Soest *et al*. [18] and adapted for the Ankom^200^ Fiber Analyzer (Ankom Technology, Macedon, NY). Total digestible nutrients (**TDN**) were calculated by the following equation: TDN(%) = DCP + DNDF + DNFC + (2.25 × EE), where DCP = apparent digestible CP, DNDF = apparent digestible NDF, DNFC = apparent digestible non-fiber carbohydrates (**NFC**), and DEE = apparent digestible EE. The NFC were calculated as NFC = 100 − [(CP − CP_from urea_ + Urea) + NDF + EE + Ash] [12].

### Statistical Analysis

Experimental units, defined as the smallest unit upon which a measure was made, were selected according to Robinson *et al*. [19]. For parameters measured at the animal level (performance and carcass characteristics), animal was used as the experimental unit and pen was included as a random effect in the model. For parameters measured at the pen level (intake, digestibility, and G: F), pen was used as the experimental unit and no random effect was added in the model. All parameters were analyzed using the following model:

$${\text{Y}}_{\text{ij}}={\text{B}}_{0}+{\text{B}}_{1}{\text{X}}_{\text{i}}+{\text{B}}_{1}{\text{X}}_{\text{i}}^{2}+{\text{e}}_{\text{ij}},$$

where:

Y_ij_ is the observed measurement of the i^th^ level of CG inclusion in the diet and of the j^th^ experimental unit; i = 1, 2, 3, 4 (levels of inclusion of CG as a replacement of DGC), B_0,_ B_1_ = regression parameters of the model; X_j_ = effect of i^h^ level of fixed quantitative factor (replacement of CG with DGC); e_ij_ = residual error, assuming e_ij_ ~ N (0, s^2^). All statistical procedures were carried out using the mixed procedure of SAS 9.2 (SAS Institute Inc., Cary, NC) and significance was established at α = 0.05.

## Results

### Intake and digestibility

**Fig 1:**
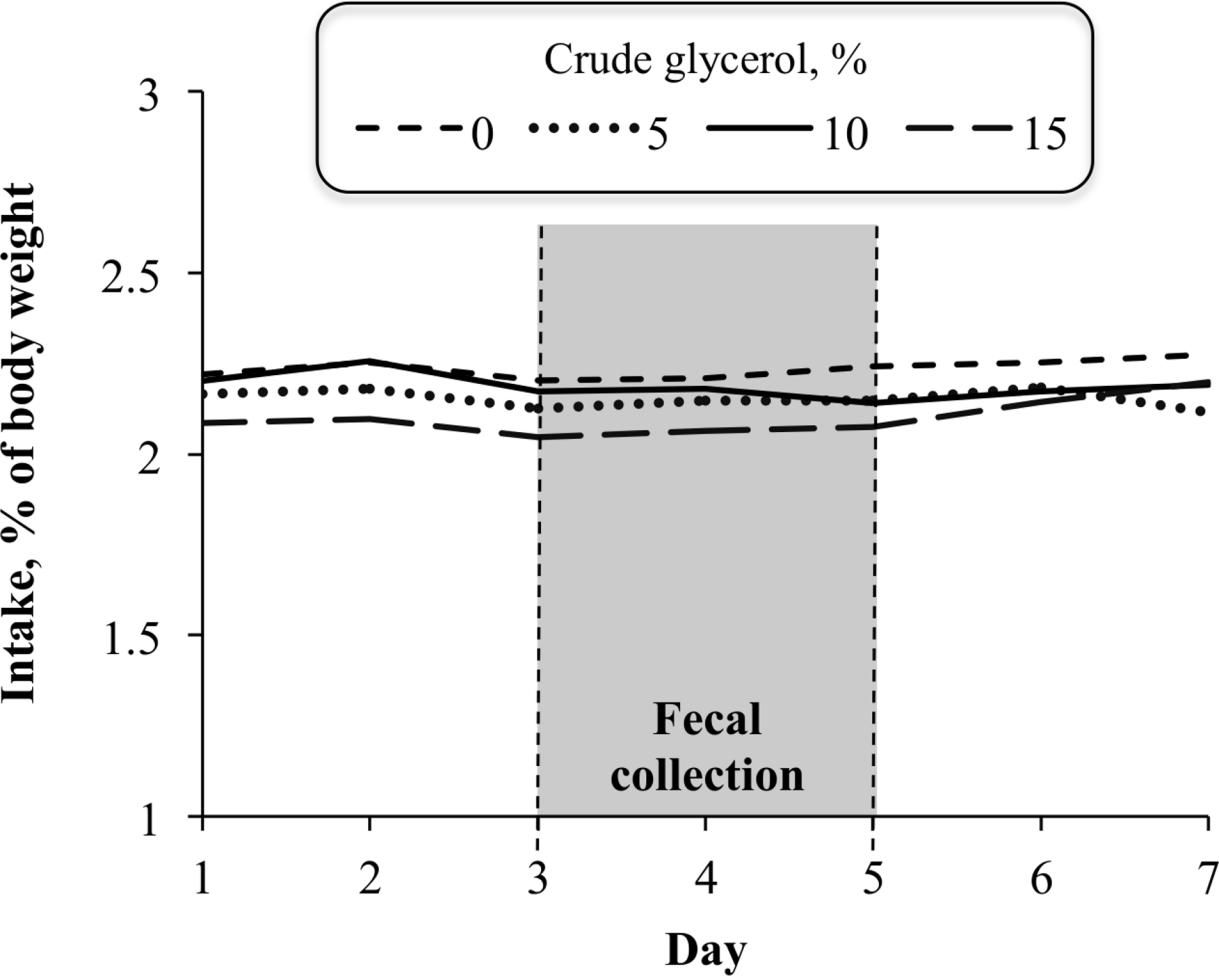
Effect of crude glycerol inclusion on average dry matter intake during fecal collection.

The inclusion of CG linearly decreased (*P* < 0.05) DM, OM, and NDF intake (Table 2). The DM intake of the control treatment (0% of CG) was about 10% greater than the treatment with 15% CG inclusion. However, CG levels did not affect (*P* > 0.05) intakes of CP, NFC, and TDN, which averaged 1.54 ± 0.09, 4.80 ± 0.40 and 7.04 ± 0.46 kg/d, respectively. The DM intake variation during the fecal collection period is presented in Fig 1.

**Table 2:** Effect of crude glycerol inclusion on daily intake of dietary nutrients in finishing beef cattle.

	Crude glycerol, %					*P*-value	
Intake^1^	0	5	10	15	SEM	Linear	Quadratic
DM, kg/d	10.5	10.2	10.2	9.6	0.15	<0.01	0.60
DM, % BW	2.40	2.36	2.33	2.23	0.14	<0.01	0.42
OM, kg/d	9.77	9.38	9.39	8.78	0.15	<0.01	0.54
CP, kg/d	1.59	1.57	1.52	1.50	0.02	0.09	0.98
NDF, kg/d	2.85	2.55	2.47	2.42	0.05	<0.01	0.09
NFC^2^, kg/d	4.97	4.80	4.99	4.46	0.09	0.10	0.31
TDN^3^, kg/d	7.35	6.82	7.06	7.04	0.12	0.63	0.21

^1^DM = dry matter; OM = organic matter; CP = crude protein; NDF = neutral detergent fiber; NFC = non-fiber carbohydrate; TDN = total digestible nutrients.

^2^NFC = 100 − [(CP − CP from urea + urea) + NDF + EE + Ash] [12].

^3^TDN = DCP + DNDF + DNFC + (2.25 × DEE), where DCP = apparent digestible CP, DNDF = apparent digestible NDF, DNFC = apparent digestible non-fiber carbohydrates, and DEE = apparent digestible EE.

**Fig 2:**
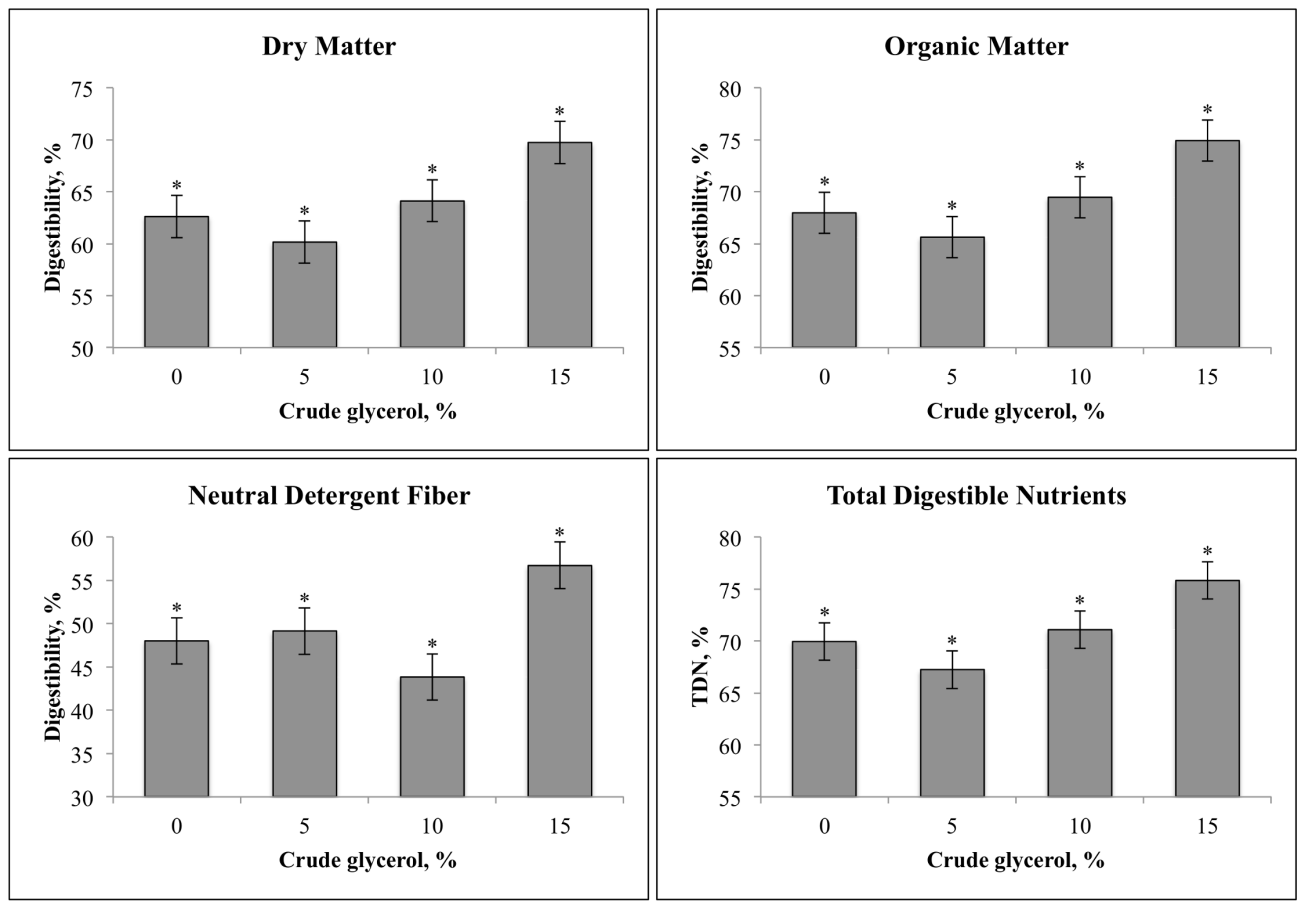
Effect of crude glycerol inclusion on apparent digestibility of dietary nutrients in finishing beef cattle. *Quadratic effect (P < 0.01).

**Fig 3:**
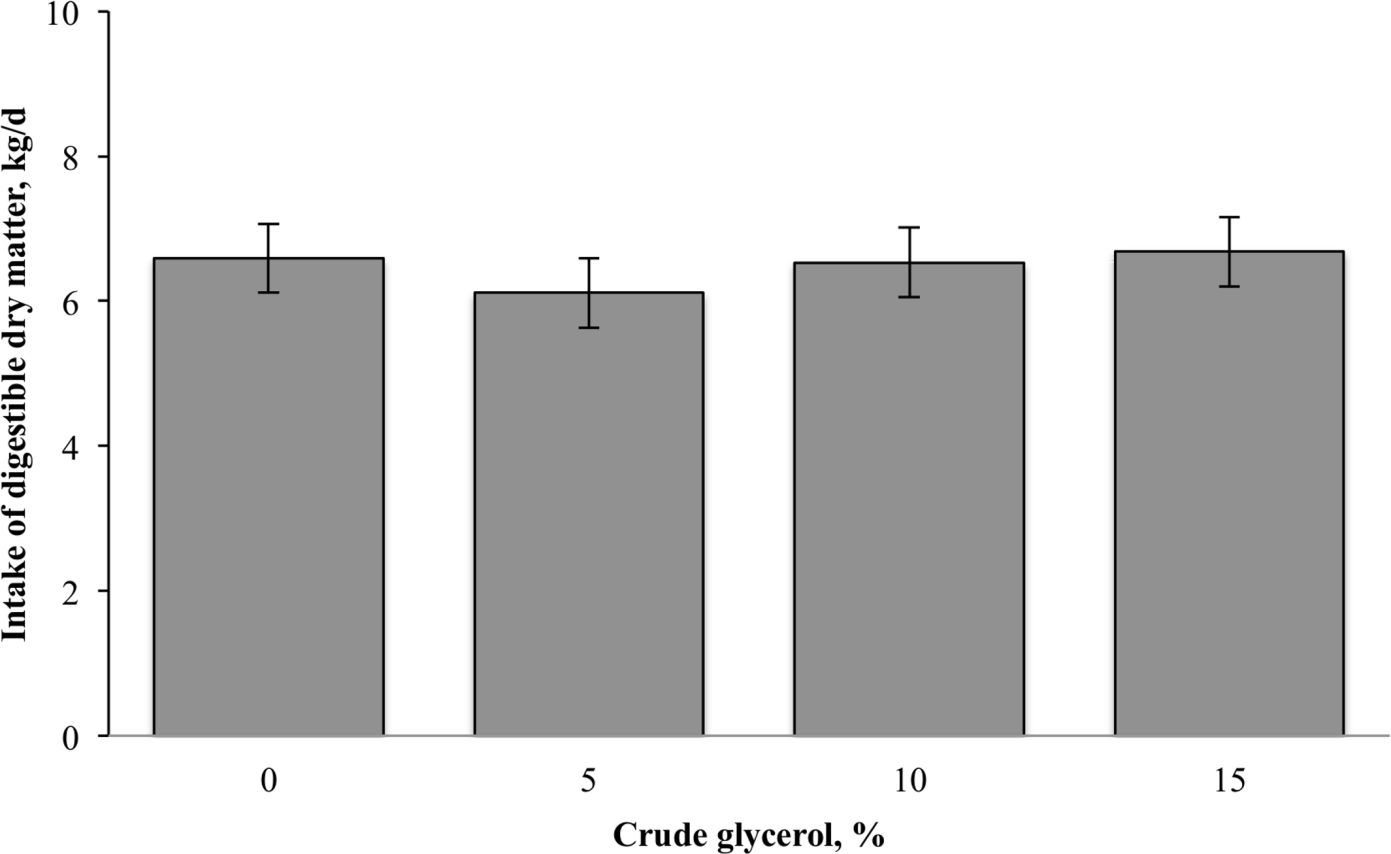
Effect of crude glycerol inclusion on intake of digestible DM in finishing beef cattle.

A quadratic effect was observed (*P* < 0.05) for digestibility traits (Fig 2). The digestibility of DM, OM, and TDN decreased when 5% CG was included in the diet compared with the control diet and increased when 10 and 15% CG was included in the diet (Fig 2). Digestibility of NDF decreased when 10% CG was included in the diet compared with 5% CG inclusion and increased when 15% CG was included in the diet compared with 10% CG inclusion. However, the inclusion of CG did not affect (*P* > 0.05) the intake of digestible DM (Fig 3), which averaged 6.48 ± 0.48 kg/d.

### Performance and Carcass Characteristics

No differences were observed (*P* > 0.05) for initial BW (367.0 ± 36.8 kg), final BW (502.3 ± 38.5 kg), ADG (1.37 ± 0.29 kg/d), carcass ADG (0.85 ± 0.23 kg/d), gain-to-feed ratio (**G:F**; 0.136 ± 0.014 kg/kg), and carcass G:F (0.857 ± 0.076 kg/kg) (Table 3).

**Table 3:** Effect of crude glycerol inclusion on performance of finishing beef cattle.

	Crude glycerol, %					*P*-value	
Item^1^	0	5	10	15	SEM	Linear	Quadratic
Body weight, kg							
Initial	370	363	372	363	0.636	0.89	0.96
Final	510	499	504	497	2.178	0.20	0.45
Performance							
ADG, kg/d	1.41	1.37	1.35	1.34	0.025	0.20	0.78
G:F, kg/kg	0.13	0.14	0.13	0.14	0.002	0.46	0.56
Carcass ADG, kg	0.89	0.85	0.84	0.83	0.019	0.23	0.67
Carcass G:F, kg/kg	0.09	0.08	0.08	0.09	0.001	0.56	0.15

^1^ADG = average daily gain

G:F = gain-to-feed ratio

Carcass characteristics are presented in Table 4. Initial carcass weight (199.2 ± 19.9 kg), initial LMA (53.9 ± 6.17 cm^2^), initial BFT (1.45 ± 0.29 mm), and initial RFT (1.56 ± 0.61 mm) were not different among treatments (*P* > 0.05). Inclusion of CG did not affect (*P* > 0.05) final carcass weight (284.5 ± 24.7 kg), carcass dressing (56.7 ± 3.23%), final LMA (81.9 ± 7.99 cm^2^), LMA gain (27.9 ± 8.04 cm^2^), final BFT (4.48 ± 1.53 mm), BFT gain (3.02 ± 1.51 mm), final RFT (6.44 ± 1.90 mm), and RFT gain (4.90 ± 1.76 mm).

**Table 4:** Effect of crude glycerol inclusion on carcass characteristics of finishing beef cattle.

	Crude glycerol, %					*P*-value	
Item^1^	0	5	10	15	SEM	Linear	Quadratic
HCW, kg	290	282	285	281	1.44	0.07	0.35
Dressing, %	56.9	56.7	56.6	56.5	0.33	0.64	0.92
LMA, cm x cm							
Initial	54.0	53.7	53.6	54.9	0.46	0.58	0.37
Final	83.0	82.2	82.1	80.9	0.40	0.06	0.88
LMA gain	28.8	28.4	28.5	25.9	0.61	0.11	0.34
Back fat, mm							
Initial	1.44	1.44	1.43	1.48	0.01	0.31	0.39
Final	4.62	4.37	4.44	4.51	0.09	0.14	0.12
Back fat gain	3.18	2.92	3.00	3.02	0.05	0.45	0.10
Rump fat, mm							
Initial	1.53	1.56	1.55	1.60	0.02	0.29	0.39
Final	6.65	6.39	6.42	6.39	0.06	0.09	0.25
Rump fat gain	5.11	4.83	4.86	4.80	0.09	0.08	0.30

^1^HCW = hot carcass weight

LMA *= Longissimus thoracis* muscle area

### Comparison Between the Two Types of Carcass Classification: Abattoir vs. Ultrasound

**Fig 4:**
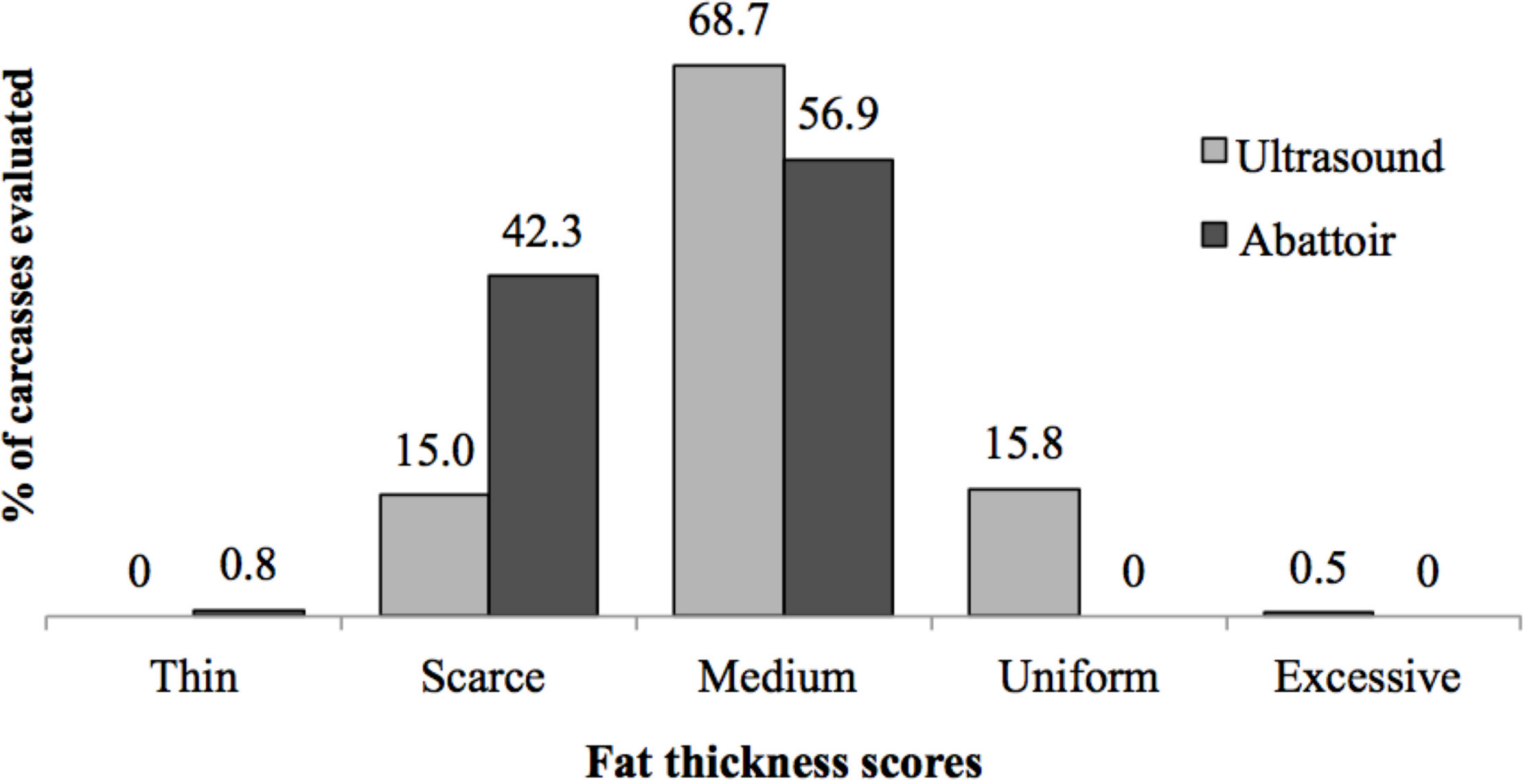
Beef cattle carcass classification by two different methods (ultrasound and abattoir). Fat cover classified as thin (less than 1 mm), scarce (1–3 mm), medium (3–6 mm), uniform (6–10 mm) and excessive (over 10 mm).

The comparison between the two types of carcass classification (abattoir *vs*. ultrasound) is presented in Fig 4. The abattoir scores classified 0.8% of the carcasses as thin, 42.3% scarce, and 56.9% medium fat. The ultrasound analysis classified 15% of the carcasses as scarce, 68.7% medium fat, 15.8% uniform fat, and 0.5% excessive fat. Comparing the two methods of carcass evaluation, it was found that generally, ultrasound analysis overestimated fat deposition when compared to the abattoir classification, 65% of the carcasses classified as scarce fat by the abattoir were classified as medium fat in the ultrasound analysis, as well as 28% of the carcasses classified as medium fat by the abattoir were classified as uniform fat in the ultrasound analysis.

## Discussion

### Intake and Digestibility

We hypothesized that partial replacement of DGC with CG would not compromise DM intake and apparent digestibility. However, compared to DGC, CG decreased intakes of DM, OM, and NDF in finishing beef cattle diets. Nevertheless, the intakes of CP, NFC, and TDN were not affected by the inclusion of CG. The lack of effect observed on TDN intake indicates an increase in energy density of the diets as CG replaced DGC. Mach *et al*. [3] estimated glycerol metabolic energy at 4.03 Mcal/kg. In this study the estimated CG (90.38% of glycerol, DM basis) metabolic energy was 3.64 Mcal/kg, which is 12% greater than the 3.25 Mcal/kg of corn [10]. Monnerat *et al*. [4] also observed higher energy levels for CG compared to corn, suggesting that CG contributes with more energy per unit of DM than corn. That would explain why in this study the intake of DM decreased with CG inclusion. Also, the inclusion of CG did not affect (*P* > 0.05) the intake of TDN and digestible DM in this study, showing that energy intake was not influenced by CG inclusion. Diets with greater CG levels had lower NDF levels and this may explain the reduction on NDF intake with CG inclusion.

Reduction in DM intake has been observed in cattle fed CG and the literature suggests possible reasons for this observation ranging from effects on ruminal metabolism up to effects on intermediary metabolism. Parsons *et al*. [20] suggest that concentrations above 5% in the diets may impair rumen function, resulting in reduced DM intake. These authors observed a linear decrease on DM intake in crossbred heifers fed finishing diets containing 0, 2, 4, 8, 12, or 16% CG (DM basis). Pyatt *et al*. [21] replaced cracked corn with CG up to 10% in finishing diets of Angus-crossbred steers resulting in a decrease of 10% on DM intake and increase of 11.4 and 19.2% on ADG and feed efficiency, respectively. Also, Hales *et al*. [22] observed a linear reduction in DM intake of steers when CG replaced roughage at 0, 2.5, 5, and 10% of the diet. Trabue *et al*. [23], using an *in vitro* system, reported that lactic acid concentration increased rapidly through the first 8 h of incubation and according to these authors, this may have slowed microbial activity and glycerol fermentation, which affected DM intake. It is well known that CG has the capacity of increasing propionate concentration in the rumen [5,9,24]. When infused in isocaloric amounts in lactating cows, propionate resulted in lower DM intake than acetate [25]. Conversely, other studies have reported that DM intake was not affected by CG inclusion. Mach *et al*. [3] observed no effect on DM intake, ADG or G:F in Holstein bulls. Daily intake was not affected when four CG levels (0, 4, 8, and 12% of concentrate DM) were fed to the animals. Likewise, Eiras *et al*. [26] did not observe a reduction on DM intake when CG was added up to 17.8% in finishing diets for Purunã bulls.

The quadratic increase in DM apparent digestibility observed in animals fed diets with higher levels of CG was not expected, and the three main factors that could explain these results are: 1) The quick microbial adaptation and fast disappearance of CG in the rumen [5,27,28]. According to Donkin [9], glycerol is fermented to short chain fatty acids inside the rumen and may also be integrally absorbed through the ruminal epithelium such that 50 to 80% of the glycerol disappears within 4 hours. Furthermore, other studies observed higher volatile fatty acid concentrations in the rumen of animals fed CG [3,27,29]. Moreover, Hales *et al*. [22] observed an increase in starch digestibility when steers were fed diets containing 0, 2.5, 5, and 10% CG. This could also explain the higher values of OM and NFC digestibility in diets with the highest levels of CG; 2) The inclusion of CG decreases the acetate: propionate ratio in the rumen [6,23,28,29]. Compared to acetate, propionate synthesis in the rumen is more energy efficient [30]; therefore, this could potentially increase energy efficiency due to an increased energy availability; 3) Diets with higher levels of CG may have longer retention times in the rumen. Digestibility is the result of the competition between the rates of digestion and passage, and passage rate positively correlates with DM intake [31]. Although DM intake was lower as CG was included in the diet, feed could have remained longer in the rumen and consequently allowed greater microbial and enzymatic activity, thereby increasing DM digestibility. The increase on DM digestibility affected the intake of digestible DM, which did not differ between treatments in the present study.

Effects of CG on nutrient digestibility in ruminants have been conflicting and the variation in the chemical composition as well as the amount of CG used in different studies may be an explanation for this inconsistency. Hales et al. [32] also reported a quadratic effect in DM digestibility when CG (82.7% glycerol) was included at up to 15% (DM basis) in finishing diets. Eiras *et al*. [26] observed an increase in digestibility of DM, OM, NDF, and CP when CG (81.2% glycerol, 2.3% water, 4.8% ash, and 3.3% methanol) was added at 60, 120, and 178 g/kg of DM in beef cattle finishing diets. Moreover, Wang *et al*. [28] observed an increase in digestibility of OM, NDF, and CP when Simmental steers were fed 100, 200, and 300 g/d of CG (99% glycerol). Hess *et al*. [33] observed that DM and NDF digestibility were not affected when CG was added at up to 15% of DM in an in vitro system. Others have observed a reduction in digestibility when feeding CG. Shin *et al*. [34] noted a decrease in NDF digestibility when Holstein cows were fed diets containing 0, 5, or 10% CG (80.3% glycerol, 12.4% water, and 0.46% methanol). Furthermore, van Cleef *et al*. [35] noted a decrease in NDF digestibility when Nellore bulls were fed diets containing 0, 7.5, 15, 22.5, and 30% CG (86% glycerol, 5% water, and >0.01% methanol). These results suggest suppression in fiber digestibility when CG is included in cattle diets. Roger *et al*. [36] observed inhibition in growth and activity of two cellulolytic bacteria, *Ruminococcus flavefaciens* and *Fibrobacter succinogenes*, and of an anaerobic fungal species, *Neocallimastix frontalis* inoculated with glycerol at a concentration of 5%. Therefore, the inhibition of cellulolytic activity could affect fiber digestion, passage rate and consequently decrease intake. However, in the present study an increase (P < 0.05) was observed in NDF digestibility for treatments with 5 and 15% CG inclusion. Therefore, it is likely that other aspects such as grain processing methods, roughage source and amount, and feed additives may play a role in glycerol utilization and its effects on intake and digestibility.

### Cattle Performance and Carcass Characteristics

We hypothesized that CG may partially replace DGC and may be included at concentrations up to 15% (DM basis) without compromising performance and carcass characteristics. As expected, the replacement of DGC with CG did not change ADG, G:F, and final BW. These results are consistent with findings in previous reports and are usually related with the quality of the CG [3,35,37]. The CG used in this study had a high purity (above 80%) with low fatty acid and methanol concentration, which allowed similar performance when compared to DGC. Furthermore, the diets with higher levels of CG presented higher digestibility. In this study, the increase in digestibility observed when animals were fed higher levels of CG appeared to offset the decrease in intake and as a consequence, bulls fed higher levels of CG achieved the similar performance as bulls fed DGC. Similar results were observed by Parsons *et al*. [20] when DM intake decreased without affecting ADG in an experiment that heifers were fed up to 12% CG (DM basis). Bartoň *et al*. [38] also noted no differences in ADG and feed conversion ratio when beef bulls were fed diets containing 0, 4.7, or 9.3% of CG (DM basis). Moreover, Ramos and Kerley [37] observed no differences in growth performance of crossbreed steer calves when CG was included in the diet at up to 20% of DM. Other studies indicated that CG inclusion in the diet improved animal performance when included at up to 7.5% [39], 10% [21] and 14.9% [40] in beef cattle diets. The lack of effect observed for carcass ADG and carcass G:F demonstrates that diets containing up to 15% CG may promote the same efficiency of diet utilization than DGC diets.

Partial dietary replacement of DGC with CG did not affect hot carcass weight, carcass gain, carcass percentage dressing, LMA, BFT, and RFT traits in this study. These results were expected and as no significant differences were observed between energy intake, protein intake, and performance in the treatments, lack of effect on these variables is justified. Similarly to our results, no effects on carcass characteristics have been reported when CG was included at up to 10% in diets of finishing bulls [38,41,42]. Eiras et al. [43] also observed a lack of effects in fat thickness and LMA when CG (81.2% glycerol) was included at up to 18% (DM basis) in diets of young Purunã bulls finished in feedlot. Moreover, improved hot carcass weight, LMA, and BFT were observed when diets containing 12 and 15% CG were fed to bulls [44] and beef calves [40], respectively. As previously discussed, the glycerol can be converted to propionate in the rumen or be directly absorbed through the rumen wall [5,9], providing greater available energy to the animals. The LMA has a positive correlation with the carcass edible portion [45]. The lack of difference in LMA in this study suggests that the inclusion of CG at up to 15% in the diet has the same efficiency of DGC to support muscle growth in feedlot finishing cattle. Furthermore, the lack of difference for BFT and RFT traits suggests that CG allows a satisfactory carcass thickness for finishing bulls.

### Comparison Between the Two Types of Carcass Classification: Abattoir vs. Ultrasound

Accuracy differences in abattoir and ultrasound classifications may explain the differences observed in this study. Ultrasound evaluations give an accurate and repeatable measurement of carcass fat in beef cattle [46,47], and are an adequate method to determining carcass merit [48]. On the other hand, the abattoir system, which is the major carcass grading system used in Brazil, is subjective and less accurate. In this study, 46% of all abattoir scores were lower than ultrasound scores, which may lead to unfair financial compensation to beef producers. In situations that carcasses are awarded or penalized according to fat deposition, ultrasound analysis should be used for a more accurate evaluation of beef cattle carcasses.

## Conclusions

Results from this large-scale study indicate that CG may partially replace corn and may be included at up to 15% in finishing beef cattle diets without affecting performance and carcass characteristics. The information presented here has direct practical implications in the field. It is important not only from the performance perspective but also from a sustainable perspective since glycerol is a biofuel residue and could potentially partially replace corn as an energy source for finishing beef cattle diets.

## Supporting Information

S1 Fig Effect of crude glycerin inclusion on sensitive analysis of the cost per carcass gain as a function of crude glycerin prices in relation to ground corn prices. (TIF)

S1 Table Raw Data. (XLSX)

S1 Text Economic Analysis. (DOCX)

## Abbreviations

ADG: average daily gain
BW: body weight
BFT: back fat thickness
CG: crude glycerol
CP: crude protein
DGC: dry ground corn
DM: dry matter
EE: ether extract
G:F: gain-to-feed ratio
LMA: *Longissimus thoracis* muscle area
NDF: neutral detergent fiber
NFC: non-fiber carbohydrate
OM: organic matter
RFT: rump fat thickness
TDN: total digestible nutrients
