# Supplementary figures and images for: Partial Replacement of Ground Corn with Glycerol in Beef Cattle Diets: Intake, Digestibility, Performance, and Carcass Characteristics
Source: PLoS One. 2016 Jan 28;11(1):e0148224. doi: 10.1371/journal.pone.0148224 (PMC4731075; doi:10.1371/journal.pone.0148224)

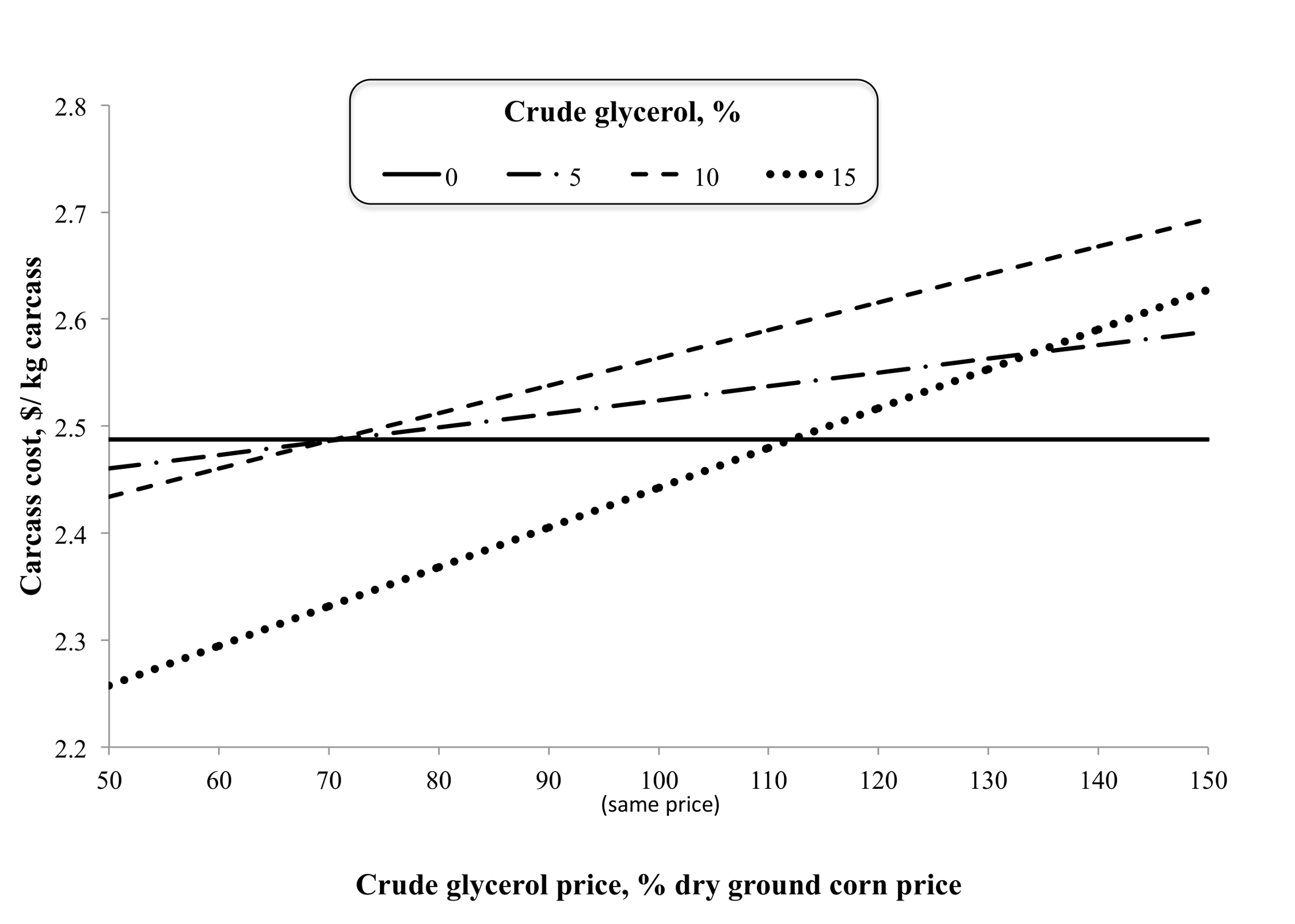

Supplement: S1 Fig — (TIF) [file pone.0148224.s001.tif]
