# Supplementary material for: Partial Replacement of Ground Corn with Glycerol in Beef Cattle Diets: Intake, Digestibility, Performance, and Carcass Characteristics
Source: PLoS One. 2016 Jan 28;11(1):e0148224. doi: 10.1371/journal.pone.0148224 (PMC4731075; doi:10.1371/journal.pone.0148224)
Supplement: S1 Text — (DOCX) [file pone.0148224.s003.docx]

# Economic Analysis

#### Material and Methods

Economic values included: diet cost ($/kg DM), animal cost ($/animal/day) and carcass cost ($/kg carcass). Feedstuff prices were obtained from market prices practiced in the state of Mato Grosso do Sul, Brazil during the period of July to November 2012. Carcass cost was calculated by dividing animal cost by carcass gain. Because feedstuff prices varies substantially over time, a sensitivity analysis was performed, according to Diniz et al. [1], to determine which diet was more economical based exclusively on the relationship between CG price as a function of DGC price.

#### Results

Treatment without CG had the lowest diet cost, followed by treatments with 5%, 10%, and 15% of CG inclusion, respectively (S1 Table). Treatment without CG had the lowest animal and carcass costs, followed by treatments with 5%, 15%, and 10% of CG inclusion, respectively (S1 Table). As expected, sensitive analysis (S1 Fig.) indicated that the lowest carcass cost changed depending on the CG: DGC price relationship. When CG price was up to 110% of DGC price, the treatment that provided the lowest carcass cost was 15% CG inclusion. However, when CG price was equal or higher than 120% of DGC price, the diet without CG was the most economical treatment.

S1 Table. Effect of crude glycerol inclusion on economic analysis.

| Item^1^ | Crude glycerol, % | | | |  |
| --- | --- | --- | --- | --- | --- |
|  | 0 | 5 | 10 | 15 | $/kg DM |
| Corn silage | 15.4 | 15.4 | 15.4 | 15.4 | 0.09 |
| Dry ground corn | 38.9 | 33.7 | 28.6 | 23.4 | 0.21 |
| Crude glycerol | 0.0 | 5.0 | 10.0 | 15.0 | 0.38 |
| Citrus pulp | 25.0 | 25.0 | 25.0 | 25.0 | 0.13 |
| Cottonseed cake | 16.6 | 16.6 | 16.6 | 16.6 | 0.23 |
| Urea | 0.80 | 0.97 | 1.13 | 1.29 | 0.55 |
| Slow release urea^2^ | 0.45 | 0.45 | 0.45 | 0.45 | 1.78 |
| Vitamin-mineral premix | 2.93 | 2.93 | 2.93 | 2.93 | 1.10 |
| Diet cost, $/kg DM | 0.21 | 0.22 | 0.23 | 0.24 |  |
| Animal cost, $/animal/d | 2.22 | 2.23 | 2.32 | 2.27 |  |
| Carcass cost, $/kg carcass | 2.49 | 2.62 | 2.76 | 2.72 |  |

¹Corn silage = $ 38.31/ton NM; Dry ground corn = $ 186.21/ton NM; Crude glycerol = $ 328.91/ton NM; Citrus pulp = $ 114.20/ton; Cottonseed cake = $216.01/ton NM; Urea = $535.30/ton NM; Slow release urea = $1,673.53/ton NM; Vitamin-mineral premix. = $ 1078.70/ton NM. Feedstuff prices were obtained from July to November of 2012.

^2^Optigen 1200 controlled-release nitrogen, Alltech, Araucária, PR, Brazil.

**S1 Fig. Effect of crude glycerol inclusion on sensitive analysis of carcass cost as a function of crude glycerol price compared to dry ground corn price.**

#### Discussion

We hypothesized that CG could partially replace DGC at up to 15% in finishing beef cattle diets without compromising production cost. However, the treatment without CG had the lowest diet cost, which can be explained by the high values paid for CG compared to DGC. The CG used in this study was imported from Rio Grande do Sul state, which is over 300 km away from the experimental site. The shipping cost substantially increased overall CG cost, which was about 180% of DGC cost. This resulted in higher diet cost observed in the CG treatments. Treatment with 15% CG inclusion had a slightly lower animal and carcass costs than treatment with 10% CG due to the lower DMI observed in the 15% CG treatment.

In this study, the relationship between CG and DGC prices was the major factor defining the most economical diet. Sensitive analysis is a technique used to determine how different values of an independent variable will impact a particular dependent variable under a given set of assumptions (i.e. glycerol price as a function of corn price impacting carcass cost). This technique is used within specific boundaries that will depend on one or more input variables, such as the effect that changes in feedstuff prices will have on carcass cost. The sensitivity analysis indicated that CG inclusion at 15% of the diet may promote a lower carcass cost when CG price is up to 110% of DGC price. Data from the sensitivity analysis indicate that CG utilization may be an economical alternative to DGC when CG price is up to 110% of DGC price.

#### References

1. Diniz L, Valadares Filho S, Campos J, Valadares R, Silva L, et al. (2010) Effects of castor meal on the growth performance and carcass characteristics of beef cattle. Asian-Aust J Anim Sci 23: 1308-1318.
